# Effect of barbed suture vs. conventional suture on postoperative recovery and uterine integrity in patients undergoing laparoscopic myomectomy

**DOI:** 10.3389/fsurg.2025.1679965

**Published:** 2025-11-04

**Authors:** Lixin Fan, Fengjiao Hu, Jingwei Zhang, Yanlei Gao, Fei Kang, Qian Zhao

**Affiliations:** 1 Obstetrics Clinic, Shijiazhuang Maternity & Child Healthcare Hospital, Shijiazhuang, Hebei, China; 2 No. 22 Haidian Cadres’ Sanatorium of Beijing Garrison, Chinese People’s Liberation Army, Beijing, China; 3 Department of Radiology, Shijiazhuang Maternity & Child Healthcare Hospital, Shijiazhuang, Hebei, China; 4 Department of Internal Medicine, Shijiazhuang Maternity & Child Healthcare Hospital, Shijiazhuang, Hebei, China

**Keywords:** laparoscopic myomectomy, barbed suture, uterine integrity, postoperative recovery, interaction

## Abstract

**Background:**

Uterine fibroids are common benign tumors in women, and laparoscopic surgery is one of the main treatment methods. The choice of suturing technique can influence postoperative recovery and uterine integrity.

**Methods:**

This retrospective study included 210 patients who underwent laparoscopic myomectomy, divided into a barbed suture group (*n* = 105) and a conventional suture group (*n* = 105) based on the suturing method. Univariate analyses compared demographic characteristics, clinical features, postoperative recovery indicators, uterine integrity assessments, wound complications, and laboratory parameters between the two groups. Multivariate logistic regression was used to evaluate the effects of suture method and baseline characteristics on uterine integrity, as well as interactions between suture method and patient factors. Quality of life scores at 1, 3, and 6 months postoperatively were also analyzed.

**Results:**

The barbed suture group showed significantly better postoperative recovery and uterine integrity indicators compared to the conventional suture group (*P* < 0.05). Although the incidence of wound complications was lower in the barbed suture group, the difference was not statistically significant. On postoperative day 2, the barbed suture group had lower white blood cell counts (*P* = 0.039), higher hemoglobin levels (*P* = 0.029), lower pain scores (*P* < 0.001), and higher SF-36 quality of life scores (*P* < 0.001). Multivariate regression analysis revealed that history of abortion, number of fibroids, and menopausal status significantly affected uterine integrity, and barbed sutures significantly improved postoperative uterine integrity (OR = 3.984, *P* = 0.019). Significant interactions existed between suture method and history of abortion as well as fibroid degeneration, with barbed sutures having a more pronounced effect on uterine integrity in patients without abortion history and fibroid degeneration. Quality of life scores during postoperative follow-up were significantly higher in the barbed suture group.

**Conclusion:**

Barbed suturing is superior to conventional suturing in laparoscopic myomectomy, effectively promoting postoperative recovery, improving uterine integrity, and enhancing long-term quality of life. Its benefits are more pronounced in patients without a history of abortion and without fibroid degeneration. Further large-scale randomized controlled trials are warranted to validate these findings and explore underlying mechanisms.

## 1 Introduction

Uterine fibroids is the most common benign tumor in women, with a high incidence rate, especially in women of childbearing age (1, 2). Although most patients with uterine fibroids remain asymptomatic, some may experience menstrual irregularities, abdominal pain, infertility, or other symptoms depending on the fibroids' size, location, or associated complications (3). The treatment methods for uterine fibroids mainly include drug therapy, surgical treatment, and interventional treatment (4, 5). Among them, laparoscopic surgery has become an important means of treating uterine fibroids due to its small trauma, fast recovery, and few complications (6, 7). However, the integrity of the uterus after surgery, the quality of scars, and the patient's postoperative recovery are still important factors affecting the surgical outcome and the patient's quality of life.

The choice of suture method in surgical procedures not only affects postoperative recovery, but also has a significant impact on the quality of wound healing, incidence of complications, and long-term quality of life of patients. Conventional suturing facilitates healing by approximating tissue edges, but it can create considerable tension on the tissue, potentially compromising blood flow and increasing the risk of scar formation. In addition, traditional suturing operations require high precision, which may lead to longer surgical time and unstable results. Barbed suture is a new type of suture with a self-locking mechanism, which means that the design of the suture with spikes can reduce the pulling on the tissue during the suture process, making the suture more stable (8). The unique characteristics of this suture thread make tissue connections tighter, while reducing tissue damage and wound tension during surgery, thereby reducing the risk of postoperative complications (9).

Currently, some studies have shown that barbed sutures are more effective in promoting postoperative recovery in patients with laparoscopic uterine fibroids compared to traditional sutures (10–12). However, research on whether barbed sutures can effectively reduce wound complications, improve uterine integrity, and their effects in different populations is relatively scarce. This study aims to fill this gap by retrospectively analyzing the clinical effects of different suture methods, exploring the potential advantages of barbed suture in uterine fibroid surgery, and providing strong evidence support for clinical practice.

## 2 Materials and methods

### 2.1 Study population

This retrospective study included patients who underwent laparoscopic myomectomy at our hospital between January 2021 and June 2024. Inclusion criteria were: (1) age ≥18 years; (2) uterine fibroids classified as international federation of gynecology and obstetrics (FIGO) Type 4, Type 5, Type 6, or Type 7, with a scheduled laparoscopic myomectomy (13); and (3) meeting surgical indications. Exclusion criteria included: (1) presence of severe systemic diseases such as malignancies or serious cardiopulmonary conditions; (2) severe hepatic or renal insufficiency; (3) significant psychiatric illness or poor compliance; and (4) missing key data (e.g., suture method). Patients were divided into two groups based on the suturing technique: the conventional suture group and the barbed suture group.

### 2.2 Surgical procedure

Under general anesthesia with endotracheal intubation, patients were placed in the lithotomy position. Pneumoperitoneum was established with intra-abdominal pressure maintained at 12 mmHg. A 1 cm skin incision was made above the umbilicus for laparoscope insertion to inspect the pelvic and abdominal cavity. Trocar access ports were inserted at the right lower abdomen (McBurney's point) and left lower abdomen (anti-McBurney's point) to establish operating channels. During the procedure, 6 units of vasopressin were injected into the uterine body to reduce bleeding. A monopolar electric hook was used to make an incision at the most prominent point of the fibroid, exposing and completely excising the fibroid. The excised fibroid was placed in a disposable specimen retrieval bag and removed by morcellation. The cavity was then thoroughly irrigated and hemostasis achieved, followed by closure of the uterine incision. All uterine incisions were sutured using a two-layer continuous suturing technique. The conventional group used absorbable Vicryl 1-0 sutures, while the barbed suture group used 30 cm 1-0 polyglyconate unidirectional barbed sutures (V-Loc 180) with a 37 mm half-circle needle.

### 2.3 Data collection

Demographic characteristics of patients were collected, including age, BMI, marital status, history of abortion, and parity. Surgical information included operative time, blood loss, and length of hospital stay. Postoperative recovery parameters included time to first ambulation and time to first flatus. Evaluation indicators of uterine integrity at 3 months postoperatively included Patient and Observer Scar Assessment Scale (POSAS) score (observer scale and patient scale), myometrial scar thickness, echogenicity of scar area, blood perfusion in the scar area, and incidence of scar niche. Wound complications, such as incision infection, wound dehiscence, hematoma, serous discharge or exudate, hypertrophic scars, or keloids, were recorded. Clinical and laboratory indicators were collected at preoperative baseline and on postoperative day 2 included white blood cell count (WBC), hemoglobin level (Hb), platelet count (PLT), C-reactive protein (CRP), Visual Analog Scale (VAS) for pain, 36-Item Short Form Health Survey (SF-36) scores, and Hospital Anxiety and Depression Scale (HADS) scores. Ultrasound was used to assess myometrial scar thickness, echogenicity, blood perfusion, and presence of scar niche. According to commonly used criteria in the literature (14), the presence of ≥2 clear blood flow signals within the scar area (Resistance Index, RI < 0.8; Peak Systolic Velocity, PSV > 10 cm/s) was defined as good perfusion; 1 intermittent blood flow signal (RI 0.8–1.0, PSV 5–10 cm/s) as poor perfusion; and no blood flow signal or only flickering dots (RI > 1.0, PSV < 5 cm/s) as absent perfusion.

### 2.4 Outcome measures

According to standards in clinical practice, good uterine integrity was defined as myometrial scar thickness ≥ 3.5 mm, homogeneous echogenicity, good blood perfusion, and absence of scar niche. Poor uterine integrity was defined as scar thickness < 3.4 mm, heterogeneous echogenicity, poor or absent perfusion, and presence of scar niche.

Myometrial scar thickness was measured at the thinnest portion using three-dimensional ultrasound; B-mode ultrasound was used to observe the echo distribution of the scar area, and color Doppler was employed to assess blood flow in the scar, reflecting local tissue viability and perfusion. Scar defects (niche) were evaluated by B-mode ultrasound to examine myometrial continuity; the presence of local discontinuity or indentation was considered indicative of a defect.

### 2.5 Statistical analysis

All statistical analyses were conducted using R software version 4.4.1. Continuous data were expressed as median (minimum–maximum), and comparisons between groups were performed using t-test or Mann–Whitney U test. Categorical data were expressed as frequency (percentage), and comparisons between groups were performed using Chi-square test. Multivariate logistic regression analysis was performed using uterine integrity (good vs. poor) as the dependent variable, with odds ratios (OR) and 95% confidence intervals (CI) calculated. A *p*-value < 0.05 was considered statistically significant.

## 3 Results

### 3.1 Differences in demographic and clinical characteristics between the barbed suture group and the conventional suture group

The results showed that the median age of all patients was 39 years (range: 29–48 years), and the median BMI was 25.4 (range: 19.6–32.3). The majority of patients were married (71.9%), and most had a history of multiple deliveries (83.81%). A total of 23.33% of patients had a history of abortion, and 10.95% had a history of infertility. Most patients were premenopausal (78.57%). Multiple fibroids were present in 74.29% of patients. The median maximum fibroid diameter was 4.5 cm (range: 2.4–6.8 cm). Most patients had FIGO Type 6 fibroids (65.71%), followed by Type 7 (17.14%), Type 5 (14.76%), and Type 4 (2.38%). Fibroid degeneration occurred in 17.14% of patients, and 7.14% had pedunculated fibroids. Baseline characteristics were relatively balanced between the two groups, with no significant differences (Table 1).

**Table 1:** Demographic and clinical characteristics differences between the barbed suture group and conventional suture group.

Variables	All patients (*n* = 210)	Barbed suture (*n* = 105)	Conventional suture (*n* = 105)	*P*-value
Age	39 (29–48)	39 (29–48)	39 (29–48)	0.387
BMI	25.4 (19.6–32.3)	25.1 (19.6–32.3)	26.2 (19.6–32.3)	0.292
Marital status				0.644
Single	27 (12.86%)	13 (12.38%)	14 (13.33%)	
Married	151 (71.9%)	77 (73.33%)	74 (70.48%)	
Divorced	28 (13.33%)	12 (11.43%)	16 (15.24%)	
Widowed	4 (1.9%)	3 (2.86%)	1 (0.95%)	
Parity				0.851
Yes	176 (83.81%)	87 (82.86%)	89 (84.76%)	
No	34 (16.19%)	18 (17.14%)	16 (15.24%)	
Abortion history				0.744
Yes	49 (23.33%)	26 (24.76%)	23 (21.9%)	
No	161 (76.67%)	79 (75.24%)	82 (78.1%)	
History of infertility				0.185
Yes	23 (10.95%)	8 (7.62%)	15 (14.29%)	
No	187 (89.05%)	97 (92.38%)	90 (85.71%)	
Menopausal status				0.346
Premenopausal	165 (78.57%)	86 (81.9%)	79 (75.24%)	
Perimenopausal	38 (18.1%)	15 (14.29%)	23 (21.9%)	
Postmenopausal	7 (3.33%)	4 (3.81%)	3 (2.86%)	
Number of fibroids				0.875
Single	54 (25.71%)	26 (24.76%)	28 (26.67%)	
Multiple	156 (74.29%)	79 (75.24%)	77 (73.33%)	
Maximum diameter of fibroid (cm)	4.5 (2.4–6.8)	4.5 (2.4–6.7)	4.5 (2.4–6.8)	0.466
International Federation of Gynecology and Obstetrics (FIGO) type				0.077
Type 4	5 (2.38%)	3 (2.86%)	2 (1.9%)	
Type 5	31 (14.76%)	20 (19.05%)	11 (10.48%)	
Type 6	138 (65.71%)	70 (66.67%)	68 (64.76%)	
Type 7	36 (17.14%)	12 (11.43%)	24 (22.86%)	
Degeneration				0.099
Yes	36 (17.14%)	13 (12.38%)	23 (21.9%)	
No	174 (82.86%)	92 (87.62%)	82 (78.1%)	
Pedunculated fibroid				0.310
Yes	15 (7.14%)	11 (10.48%)	4 (3.81%)	
No	195 (92.86%)	94 (89.52%)	101 (96.19%)	

### 3.2 Differences in postoperative recovery and uterine integrity indicators between barbed and conventional suture groups

The results indicated that the barbed suture group had significantly shorter operative time (*P* < 0.001), less intraoperative blood loss (*P* = 0.024), shorter hospital stay (*P* = 0.038), earlier time to first ambulation (*P* = 0.030), and earlier time to first flatus (*P* = 0.042). Regarding uterine integrity, patients in the barbed suture group had significantly lower POSAS scores (observer scale: *P* < 0.001; patient scale: *P* = 0.002), greater myometrial scar thickness (*P* = 0.036), more homogeneous echogenicity in the scar area (*P* = 0.006), better blood perfusion in the scar region (*P* = 0.008), and a lower incidence of scar niche (*P* = 0.010) (Table 2).

**Table 2:** Postoperative recovery and uterine integrity indicators differences between the barbed suture group and conventional suture group.

Variables	Barbed suture (*n* = 105)	Conventional suture (*n* = 105)	*P*-value
Surgical time (min)	74.0 (62.2–97.9)	85.0 (63.3–97.8)	<0.001
Blood loss (mL)	71.3 (46.0–101.8)	77.7 (46.5–101.4)	0.024
Length of hospital stay (days)	4 (3–6)	5 (3–6)	0.038
Time to first ambulation (hours)	10.8 (7.3–16.2)	12.1 (7.3–16.1)	0.030
Time to first flatus (hours)	24.4 (20.5–28.3)	25.4 (20.5–28.3)	0.042
Patient and Observer Scar Assessment Scale (POSAS)-Observer Scale	18 (12–29)	23 (12–30)	0.000
Patient and Observer Scar Assessment Scale (POSAS)-Patient Scale	23 (16–35)	28 (16–35)	0.002
Myometrial scar thickness (mm)	4.3 (3.2–5.1)	4.1 (3.2–5.1)	0.036
Echogenicity of scar area			0.006
homogeneous echogenicity	95 (90.48%)	79 (75.24%)	
heterogeneous echogenicity	10 (9.52%)	26 (24.76%)	
Blood perfusion of scar area			0.008
Good	88 (83.81%)	61 (58.1%)	
Poor	16 (15.24%)	35 (33.33%)	
Absent	1 (0.95%)	9 (8.57%)	
Scar niche			0.010
Yes	4 (3.81%)	16 (15.24%)	
No	101 (96.19%)	89 (84.76%)	

### 3.3 Differences in wound complications between the barbed and conventional suture groups

The incidence rates of incision infection, wound dehiscence, hematoma, serous discharge or exudate, hypertrophic scarring, and keloids were lower in the barbed suture group compared to the conventional group, but these differences did not reach statistical significance (all *P* > 0.05) (Table 3).

**Table 3:** Difference in wound complications between the barbed suture group and conventional suture group.

Complications	Barbed suture (*n* = 105)	Conventional suture (*n* = 105)	*P*-value
Incisional infection			0.365
Yes	1 (0.95%)	4 (3.81%)	
No	104 (99.05%)	101 (96.19%)	
Wound dehiscence			0.477
Yes	0 (0%)	2 (1.9%)	
No	105 (100%)	103 (98.1%)	
Wound hematoma			0.130
Yes	0 (0%)	4 (3.81%)	
No	105 (100%)	101 (96.19%)	
Wound seroma or exudate			0.614
Yes	1 (0.95%)	3 (2.86%)	
No	104 (99.05%)	102 (97.14%)	
Hypertrophic scar or keloid			0.477
Yes	0 (0%)	2 (1.9%)	
No	105 (100%)	103 (98.1%)	

### 3.4 Differences in clinical and laboratory indicators between barbed and conventional suture groups

At admission, there were no significant differences between the two groups in white blood cell count, hemoglobin level, or platelet count. On postoperative day 2, the barbed suture group had significantly lower WBC levels (*P* = 0.039), higher Hb levels (*P* = 0.029), higher PLT levels (*P* = 0.041), lower CRP levels (*P* = 0.027), lower VAS pain scores (*P* < 0.001), higher SF-36 scores (*P* < 0.001), lower HADS-Anxiety scores (*P* = 0.012), and lower HADS-Depression scores (*P* = 0.015) (Table 4).

**Table 4:** Difference in clinical and laboratory indicators between the barbed suture group and conventional suture group.

Variables	Barbed suture (*n* = 105)	Conventional suture (*n* = 105)	*P*-value
White blood cell count, WBC			
At admission	6.1 (4.2–8.1)	6.4 (4.2–8.3)	0.434
Postoperative Day 2	9.2 (6.6–12.8)	10.1 (6.6–12.7)	0.039
Hemoglobin, Hb (g/dL)			
At admission	13.5 (12.6–14.8)	13.6 (12.6–14.8)	0.426
Postoperative Day 2	13.1 (11.8–13.9)	12.7 (11.8–13.9)	0.029
Platelet count, PLT (×10⁹/L)			
At admission	203.7 (136.2–266.1)	194.3 (135.9–265.3)	0.588
Postoperative Day 2	175.9 (118.2–235.7)	166.8 (118.7–235.0)	0.041
C-reactive protein, CRP (mg/L)			
At admission	2.7 (1.4–4.0)	2.7 (1.3–4.1)	0.821
Postoperative day 2	20.3 (8.9–34.9)	25.2 (8.6–35.4)	0.027
VAS pain level			
At admission	2 (0–3)	2 (0–3)	0.607
Postoperative day 2	3 (2–6)	5 (2–6)	0.000
36-Item Short Form Health Survey, SF-36			
At admission	66 (60–72)	66 (60–72)	0.274
Postoperative Day 2	57 (48–61)	52 (48–61)	0.000
Hospital Anxiety and Depression Scale, HADS-Anxiety			
At admission	6 (4–7)	5 (4–7)	0.441
Postoperative day 2	8 (6–10)	9 (6–10)	0.012
Hospital Anxiety and Depression Scale, HADS-Depression			
At admission	3 (2–5)	4 (2–5)	0.171
Postoperative Day 2	6 (4–9)	7 (4–9)	0.015

### 3.5 Multivariate logistic regression analysis of the effects of suture method and baseline characteristics on uterine integrity

The results indicated that a history of abortion (OR = 0.268, *P* = 0.027), menopausal status (OR = 0.144, *P* = 0.001), a higher number of fibroids (OR = 0.759, *P* = 0.002), and fibroid degeneration (OR = 0.113, *P* = 0.001) were associated with poorer postoperative uterine integrity. Use of barbed sutures was significantly associated with better uterine integrity (OR=3.984, *P* = 0.019) (Table 5).

**Table 5:** Multivariate logistic regression analysis of the impact of suture method, baseline characteristics, and their interaction on uterine integrity.

Term	*p*.value	OR	CI_lower	CI_upper
Marital status Divorced	0.768	0.585	0.016	20.796
Marital status Married	0.945	0.890	0.032	24.946
Marital status Single	0.800	0.621	0.015	25.034
Age	0.596	0.974	0.882	1.075
BMI	0.959	1.004	0.869	1.159
Parity	0.084	0.223	0.041	1.222
Abortion history	0.027	0.268	0.084	0.860
History of infertility	0.333	0.427	0.076	2.393
Menopausal status	0.001	0.144	0.047	0.439
Number of fibroids	0.002	0.759	0.636	0.907
Size	0.346	0.818	0.538	1.242
FIGO stage	0.240	1.616	0.725	3.600
Degeneration	0.001	0.113	0.029	0.432
Pedunculated fibroid	0.128	4.616	0.643	33.126
Method	0.019	3.984	1.255	12.646
Interaction effects				
Method*Abortion history	0.038	0.162	0.029	0.907
Method*Menopausal status	0.515	0.574	0.108	3.054
Method*Number of fibroids	0.774	1.099	0.576	2.098
Method*Degeneration	0.023	0.230	0.065	0.816

### 3.6 Interaction effects between suture method and baseline characteristics on postoperative uterine integrity

The results showed a significant interaction between suture method and history of abortion (OR = 0.162, *P* = 0.038), with a negative interaction coefficient, indicating that barbed sutures had a more pronounced positive effect on uterine integrity in patients without a history of abortion, while the impact was relatively smaller in those with an abortion history. The interaction between suture method and fibroid degeneration also showed a negative effect on uterine integrity (OR = 0.230, *P* = 0.023), suggesting that barbed sutures are more suitable for patients without fibroid degeneration (Table 5).

### 3.7 Differences in postoperative quality of life at 1, 3, and 6 months between the two groups

**Figure 1:**
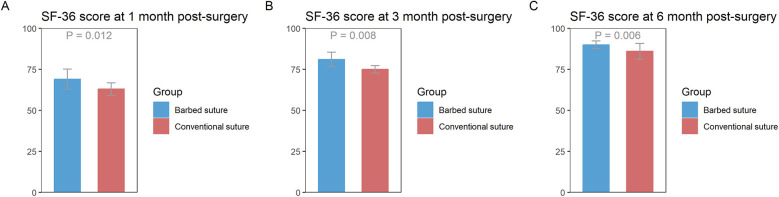
**(A)** Difference in SF-36 scores between the barbed suture group and the conventional suture group at 1 month postoperatively. **(B)** Difference in SF-36 scores between the barbed suture group and the conventional suture group at 3 months postoperatively. **(C)** Difference in SF-36 scores between the barbed suture group and the conventional suture group at 6 months postoperatively. [Data are presented as mean ± standard error (SE). *Y*-axis represents SF-36 scores (range 0–100)].

The results showed that at 1, 3, and 6 months postoperatively, the SF-36 scores in the barbed suture group were significantly higher than those in the conventional suture group (Figures 1A–C).

## 4 Discussion

Our study found that the barbed suture group had a shorter operative time, less intraoperative blood loss, and faster postoperative recovery. This may be attributed to the unidirectional or bidirectional barbs on the surface of barbed sutures, which allow them to self-anchor within tissue and eliminate the need for knot tying, thereby reducing the time spent on frequent knotting in traditional suturing (15). Moreover, conventional suturing often necessitates an assistant to maintain tension or the use of ligation instruments, whereas barbed sutures enable independent operation, streamlining the procedure and minimizing instrument changes. The barbed design distributes tension evenly, rapidly closes wounds, and minimizes tissue tearing or oozing during suturing. Conventional suturing may require electrocautery for hemostasis, whereas barbed sutures can reduce reliance on auxiliary hemostasis through tight tissue approximation. Furthermore, barbed sutures decrease tissue traction, thereby reducing tissue damage and local ischemia. The even distribution of suture tension can also reduce postoperative pain and local edema (16). If the barbed suture is made of absorbable material, there is no need for removal, further lowering the risk of infection. These factors contribute to faster postoperative recovery.

The barbed suture group demonstrated significantly better outcomes in uterine integrity metrics such as scar assessment scores, scar thickness, and blood perfusion. This may be due to the even distribution of tension, which avoids localized stress concentrations caused by knots in traditional suturing, reducing tissue ischemia and excessive scar formation. The self-anchoring nature of barbed sutures ensures tight approximation of the uterine myometrium, reduces dead space, and promotes continuous regeneration of muscle fibers, resulting in a thicker (but not pathologically thickened) scar. In contrast, overly tight knots in conventional suturing may cause local ischemia and scar contracture. Barbed sutures may facilitate more uniform myometrial healing, reducing irregularities in scar tissue and improving echogenicity and thickness. Uniform healing also helps reduce the occurrence of scar niches. The knotless design of barbed sutures reduces mechanical obstruction to blood flow, facilitates neovascularization, and lessens pressure-induced perfusion impairment, thereby enhancing blood supply to the scar tissue.

Due to the shorter operative time in the barbed suture group, the wound is exposed to air (and potential bacterial contamination) for a shorter duration, thus reducing infection rates. The uniform tension distribution aids in tight wound edge approximation, promoting healing and lowering the risk of dehiscence, hematoma, or hypertrophic scar formation due to asymmetrical closure or overly tight sutures. Better tissue approximation also reduces wound gaps, preventing fluid infiltration into surrounding areas and decreasing seroma or exudate formation. Enhanced perfusion with barbed sutures can improve local circulation, promote early healing, and reduce fluid accumulation. It should be noted that although the incidence of wound complications in the barbed suture group was lower than that in the conventional suture group, none of the differences reached statistical significance due to the limited sample size. Therefore, these results can only be cautiously interpreted as suggesting that barbed sutures may help reduce the risk of wound complications to some extent, but no definitive conclusions can be drawn. This also indicates that future studies with larger sample sizes are needed to further validate the potential advantages of barbed sutures in postoperative wound management.

A novel aspect of this study is the interaction analysis, which revealed that barbed sutures are more effective in patients without a history of miscarriage. A history of miscarriage may impair endometrial and myometrial regenerative capacity, thereby affecting uterine recovery (17, 18). In contrast, patients without miscarriage generally have better endometrial and myometrial regenerative capacity, greater myometrial elasticity, and better blood supply, which enhances the benefits of barbed sutures. Barbed sutures also showed superior performance in patients without fibroid degeneration, likely because the myometrium surrounding non-degenerated fibroids remains structurally intact with well-aligned collagen fibers, enabling the barbs to anchor more effectively in healthy tissue (19, 20). Degenerated fibroids may exhibit softening, liquefaction, or necrosis, compromising tissue stability and suture holding strength. Non-degenerated fibroids also have clearer boundaries, facilitating precise suturing and maximizing the advantages of barbed sutures, while degenerated fibroid tissue is fragile and prone to tearing, diminishing the mechanical advantages of barbed sutures.

We used myometrial scar thickness, scar echogenicity, blood perfusion, and scar defects as indicators to evaluate uterine integrity. The main reason is that myometrial scar thickness reflects the degree of reconstruction of muscle fibers and connective tissue; sufficient thickness usually indicates good myometrial continuity and higher mechanical strength. Previous studies have shown that lower uterine segment scar thickness below 3.5 mm is associated with an increased risk of uterine rupture (21), which also served as a reference for the standards set in this study. Adequate blood perfusion indicates normal tissue metabolism in the scar area, providing oxygen and nutrients, promoting tissue repair and neovascularization, accelerating wound healing, and reducing the risk of local ischemia (22). Studies have also shown that when the RI exceeds 0.8, blood flow is significantly impeded (23). According to the Color Doppler Flow Imaging (CDFI) standard, grade 0 represents no detectable blood flow, indicating local ischemia; grade 1 indicates very sparse blood flow, with insufficient perfusion; grade 2 represents moderate blood flow, with adequate perfusion; and grade 3 indicates abundant blood flow, suggesting good perfusion (24). This also served as a reference for the scar assessment criteria in the present study.

Multiple studies have shown that the laparoscopic surgery chosen in this study has advantages over open surgery, including smaller surgical trauma, less intraoperative blood loss, faster postoperative recovery, and shorter hospital stay. In addition, laparoscopic surgery provides an enlarged and clear surgical view, which helps to precisely remove fibroids, preserve uterine structure, and reduce postoperative adhesions and scar formation, which is particularly important for women wishing to preserve fertility (25). In the barbed suture group, the operative time was shorter, intraoperative blood loss was reduced, postoperative recovery was faster, and the incidence of complications was lower, suggesting that barbed sutures offer advantages in terms of convenience and safety in laparoscopic myomectomy (26). Moreover, based on the observed improvements in uterine healing with barbed sutures in this study, we speculate that they may have a positive impact on women with preserved fertility. Adequate myometrial scar thickness, uniform scar echogenicity, and good blood perfusion can help restore uterine structure and function, potentially reducing the risks of uterine rupture, placental abnormalities, and miscarriage after surgery. In addition, tight and evenly distributed suturing may reduce scar formation and adhesions, thereby optimizing the environment for embryo implantation and potentially improving pregnancy success rates. However, as this study did not follow up on pregnancy outcomes, these speculations need to be validated in future prospective studies.

In this study, accurate preoperative assessment of the nature of uterine fibroids is crucial. Although the majority of uterine fibroids are benign, a small number of cases may be uterine sarcomas, posing a certain risk of misdiagnosis, which could lead to tumor dissemination or delayed treatment (27). Therefore, preoperative imaging evaluation, necessary laboratory tests, and careful intraoperative handling are of great importance in reducing the risk of misdiagnosis. In addition to the application of barbed sutures in laparoscopic myomectomy, the continuous development of minimally invasive surgical techniques offers new possibilities for improving postoperative recovery and preserving uterine function. For example, 3D laparoscopy provides a clearer stereoscopic view, which can enhance the precision and safety of uterine surgery (28, 29). Moreover, vaginal natural orifice transluminal endoscopic surgery (vNOTES), as an emerging minimally invasive technique, offers advantages such as less trauma, faster recovery, and better cosmetic outcomes, and has shown promising results in ovarian and bladder surgery (30).

However, this study has limitations. Firstly, the retrospective design may introduce biases in patient enrollment, case sources, and time of inclusion, making it difficult to achieve complete balance in the distribution of patient characteristics. Second, the sample size is relatively small, and the study did not explore the underlying mechanisms by which barbed sutures improve uterine integrity. Other possible confounding factors, such as surgical difficulty and surgeon experience, were not considered. These factors may to some extent influence postoperative recovery, wound healing, and uterine integrity. In addition, variability in surgical details and perioperative management protocols across institutions may limit the generalizability of our conclusions. Future large-scale, multicenter randomized controlled trials combined with physiological and biochemical studies are needed to investigate the mechanisms in depth.

## 5 Conclusion

This study demonstrates that barbed sutures significantly improve postoperative recovery and uterine integrity in patients undergoing laparoscopic myomectomy. They also reduce wound-related complications and enhance long-term quality of life. Factors such as history of miscarriage, menopausal status, and fibroid number significantly affect postoperative uterine integrity. Interaction analysis suggests that barbed sutures are more effective in patients without a history of miscarriage or fibroid degeneration.

## Data Availability

The raw data supporting the conclusions of this article will be made available by the authors, without undue reservation.
